# Seroprevalence and dynamics of anti-SARS-CoV-2 antibody among healthcare workers following ChAdOx1 nCoV-19 vaccination

**DOI:** 10.1017/S0950268822000747

**Published:** 2022-04-25

**Authors:** Soma Sarkar, Shantanab Das, Kabita Choudhury, Saibal Mukherjee, Raghunath Chatterjee

**Affiliations:** 1 Department of Microbiology, NRS Medical College & Hospital, 138, A J C Bose Road, Kolkata, 700014, West Bengal, India; 2 Human Genetics Unit, Indian Statistical Institute, 203 B. T. Road, Kolkata, 700108, West Bengal, India; 3 NRS Medical College & Hospital, 138, A J C Bose Road, Kolkata, 700014, West Bengal, India

**Keywords:** Anti-SARS-CoV-2 IgG antibody stability, COVISHILED, immunoglobulin G, neutralising antibody, SARS-CoV-2 vaccine, seroprevalence

## Abstract

Health care workers (HCWs) are in a higher risk of acquiring the disease owing to their regular contact with the patients. The aim of this study is to evaluate the seroprevalence among HCWs pre- and post-vaccination. The serological assessment of anti-SARS-CoV-2 antibody was conducted in pre- and post-vaccination of first or both doses of the ChAdOx1 nCoV-19 vaccine and followed up to 8 months for severe acute respiratory syndrome coronavirus 2 (SARS-CoV-2) infection and antibody titre. The neutralising antibody was positively correlated with IgG and total antibody. IgG was significantly decreased after 4–6 months post-infection. Almost all HCWs developed IgG after 2 doses of vaccine with comparable IgG to that of the infected HCWs. A follow-up of 6 to 8 months post vaccination showed a significant drop in antibody titre, while 56% of them didn't show a detectable level of IgG, suggesting the need for a booster dose. Around 21% of the vaccinated HCWs with significantly low antibody titre were infected with the SARS-CoV-2, but a majority of them showed mild symptoms and recovered in home isolation without any O_2_ support. We noticed the effectiveness of the ChAdOx1 nCoV-19 vaccine as evident from the low rate of breakthrough infection with any severe symptoms.

## Introduction

Coronavirus disease 2019 (COVID-19) started as a regional epidemic in December 2019 in Wuhan, China, but its rapid expansion made it a global pandemic affecting almost all the countries and significant mortality [1, 2]. While every affected country has taken containment and mitigation measures, but the spread of COVID-19 is still prominent [3]. The spectrum of clinical syndromes caused by severe acute respiratory syndrome coronavirus 2 (SARS-CoV-2) ranges from asymptomatic cases to mild flu-like symptoms to severe pneumonia, acute respiratory distress syndrome (ARDS) and death. Many experts believe that unnoticed, asymptomatic cases of coronavirus infection could be the hidden source of contagion [4–7]. The health care workers (HCWs) are the frontline workforce for clinical care and are presumably exposed to a higher risk of acquiring the disease than the general population. If infected, they not only pose a risk to the vulnerable patients and the fellow HCW [8–10] but associated morbidity and mental stress also cause disruption of patient care [11].

After primary infection, IgG antibody production can be maintained for a long time in any viral infection [12]. The cell entry of SARS-CoV-2 is facilitated by the interaction of the spike (S) glycoprotein through its receptor-binding domain (RBD) to the human ACE2 (hACE2) receptor and IgG antibody develops between 6 and 15 days following the disease-onset [13]. Considering the urgent need of vaccine across the globe, 120 vaccine candidates were reported within the first 5 months of 2020 [14]. Many countries including India have initiated the vaccination drive [15]. Apart from clinical trial data, more insights on antibody production are also needed for the evaluation of the results of vaccination considering the development of protective as well as therapeutic antibodies. Amidst the slow vaccination process in India, the number of cases significantly increased during the second wave [16].

Although there is evidence on the immunological responses against SARS-CoV-2, but the time to seroconversion and the antibody levels elicited in relation to the patient profile are not fully characterised yet. Importantly, the correlation between sero-positivity or antibody levels and protection against re-infection, as well as the duration of protective immunity, remains to be elucidated.

The present study determined the overall infection prevalence to SARS-CoV-2, the prevalence of asymptomatic infections, SARS-CoV-2 antibody kinetics among the HCWs following infection. We also investigated the levels of antibody production after first and second doses of vaccination, breakthrough infection and the longevity of vaccine-induced antibody up to 8 months of post-vaccination.

## Methods

### Study population

This cross-sectional study among the HCWs was executed at a tertiary care hospital, Kolkata after obtaining approval from the Institutional Ethics Committee. Among the 3997 HCWs in the tertiary care centre, 313 HCWs (11 Administrative staff, facility managers and clerical staff, 66 Junior doctors, 38 Medical officers, 81 Nursing staff, 24 Paramedical staff and research scientists, 93 Support staff, GDA, security, kitchen staff) were randomly selected and included in the study with their informed written consent for serosurvey during November 2020 to January 2021, follow up and antibody response studies following vaccination during January 2021 to December 2021.

The subjects were divided into four groups (A, B, C and D) based on their working area, direct care to COVID-19 patient, face-to-face contact (within 1 meter) and duration of exposure to the COVID-19 patients, performed any aerosol-generating procedures or direct contact with the environment where the COVID-19 patient was cared like bed, linen, medical equipment, bathroom etc. [17]. HCWs with 5 h of cumulative exposure per day to the COVID-19 patient directly or indirectly were included in Group A. Those HCWs with 4hrs, 3hrs and 1hr cumulative exposure per day to the COVID-19 patient were included in Group B, Group C and Group D, respectively. Participants with symptoms suggestive of recent infection or positive RT-PCR test result within last 14 days were excluded from the study. A structured questionnaire comprising demographics, prior symptoms, prior COVID-19 test results, working location (COVID or Non-COVID unit), co-morbidities were also collected.

### Serological analysis of IgG and total antibody

Serum samples were prepared from the clotted blood following centrifugation for 10 min at 3000 ***g*** at room temperature. Serological analysis of IgG and total antibody (IgG, IgM and IgA) were performed using enhanced chemiluminescence technology by VitrosECiQ (Ortho Clinical Diagnostics, New Jersey, US) [18].

### Neutralising antibody sandwich ELISA

To find out whether the seropositive patients were also developing the neutralising antibody, a neutralising antibody sandwich ELISA (GenScript, USA) following manufacturer's protocol was also performed [19].

### Dynamics of IgG antibody over time

Among 313 HCWs, 104 were RT-PCR confirmed COVID-19 patients. To evaluate the dynamics of the antibody titre, all RT-PCR confirmed COVID-19 HCWs were followed up at 2 months interval for 6 months of their first antibody measurement.

### Seroreactivity after vaccination

313 HCWs, who received ChAdOx1, nCoV-19 corona virus vaccine (COVISHIELD) [20], were included in the study. Blood samples were collected from 119 HCWs twenty-one days after first dose and from 99 HCWs twenty-one days after second dose (57 HCWs were common in both doses) of ChAdOx1, nCoV-19 vaccine for anti-SARS-CoV-2 antibody testing. Among these vaccinated individuals, we followed up 153 HCWs until December 2021 for breakthrough infection after first or second dose of vaccination.

### Statistical analyses

Descriptive as well as inferential analyses were performed using R Software [21]. A significance level of *P* ≤ 0.01 was considered unless it was specifically mentioned.

## Results

The mean age of the 313 HCWs was 37.83 (s.d. = 12.31), among them 51% were involved in direct care of the COVID-19 patients with different level of exposures. Based on their occupational risks and degree of exposures, HCWs were classified into four groups. The mean ages of all four groups were also approximately similar. Besides their co-morbidities, COVID-19 like symptoms, working in COVID unit and use of prophylactic drug was also recorded for each subject (Table 1).

**Table 1.** Demographic details of study participants

	All (*n* = 313)	Group A (*n* = 104)	Group B (*n* = 81)	Group C (*n* = 66)	Group D (*n* = 62)
Sex					
Male (%)	168 (53.6)	83 (79.8)	1 (1.2)	35 (53)	49 (79)
Female (%)	145 (46.4)	21 (20.2)	80 (98.8)	31 (47)	13 (21)
Age					
Median	36	38	40	26	41
Mean (s.d.)	37.83 (12.31)	39.71 (12.39)	40.6 (12.74)	26.89 (3.68)	42.72 (11)
Working in COVID unit (%)	161 (51.4)	35 (33.6)	45 (55.5)	43 (65.1)	38 (61.2)
No underlying illness (%)	244 (78)	81 (77.8)	56 (69.1)	61 (92.4)	46 (74.2)
HCQ prophylaxis taken (%)	86 (27.4)	12 (11.5)	21 (26)	19 (28.7)	34 (54.8)
Symptomatic (%)	119 (38)	48 (46.1)	27 (33.3)	25 (37.8)	19 (30.6)

### Serological analysis of IgG and total antibody

**Fig. 1.**
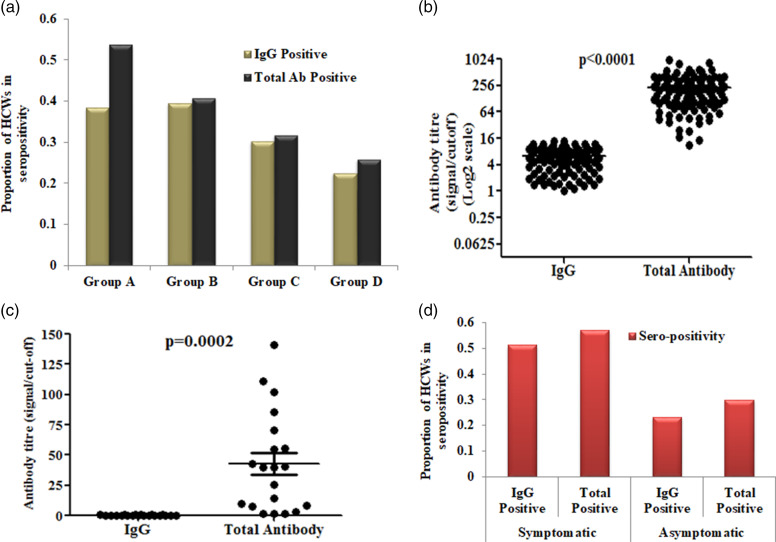
Seroprevalence among health care workers (A) Seroprevalence using anti-SARS-CoV-2 IgG and total antibody in four groups of HCWs. (B) Signal/cut off ratio of IgG and total antibody of IgG positive HCWs. (C) Signal/cut off ratio of IgG and total antibody of IgG negative HCWs. (D) Seroprevalence among COVID-19 suggestive symptomatic and asymptomatic individuals. Around 25% more symptomatic individuals developed antibody than asymptomatic individuals.

Around 34% (106) of the HCWs were seropositive for IgG while 40% (126) of them were seropositive for total antibody (IgG, IgM and IgA). The median IgG titre (signal to cut-off ratio, henceforth referred as S/Co ratio) was 6.30 with median absolute deviation (MAD) was 3.27 for IgG reactive individuals. The median total antibody titre was 141 S/Co ratio (MAD = 101.60) among sero-reactive HCWs. Seroprevalence was not significantly different with respect to age or sex of the HCWs (*P*-value = 0.4798, OR = 1.18, 95% CI = 0.74–1.88). Although the median IgG titre were comparable for four groups (median S/Co ratio for Group A = 5.48 and MAD = 3.65, Group B = 6.08 and MAD = 3.49, Group C = 6.22 and MAD = 2.68 and Group D = 7.89 and MAD = 2.86), but the seroprevalence was higher in Group A (53.8%) and gradually decreased for Group B (40.7%), Group C (31.8%) and Group D (25.8%) for total anti-SARS-CoV-2 antibody (Fig. 1A). Whereas for IgG, Group A (38.4%) and Group B (39.5%) showed similar seroprevalence and significantly lower prevalence in Group C (30.3%) and Group D (22.5%) (Fig. 1A). We further investigated the total antibody titres among IgG reactive and non-reactive individuals. The level of total antibody among IgG reactive HCWs was significantly higher compared to the level of IgG, suggesting the presence of IgM and IgA (Fig. 1B). Total antibody among IgG non-reactive HCWs varied from 0 to 140 S/Co ratio (Fig. 1C). Around 35% HCWs, who worked in the COVID-19 unit were seropositive. A similar proportion of seropositive HCWs were also observed in non-COVID unit HCWs (*P*-value = 0.9154, OR = 1.08, 95% CI = 0.64–1.63). Following ICMR guidelines [22], HCWs were advised to consume hydroxychloroquine (HCQ) as a prophylactic medication. In our entire study population only 27% have consumed HCQ as prophylaxis. The seropositivity among HCQ consumer and non-consumer was not significantly different (*P*-value = 0.3285, OR = 0.776, 95% CI = 0.44–1.30) (Table 2). As consumption of HCQ did not significantly affect the entire population, we didn't pursue it for further analysis on group-wise effect of HCQ.

**Table 2.** Association of COVID-19 symptoms with SARS-CoV-2 infection (Significant associations are highlighted in bold)

Total sample		
Factors	Odds ratio (95% CI)	*P* value
Sex	1.18 (0.74–1.88)	0.4798
COVID-19 suggestive symptoms	**3.49** (**2.14–5.70)**	**3.05 × 10^−7^**
Fever and headache	**3.39** (**2.05–5.62)**	**1.18 × 10^−6^**
GI upset	**2.88** (**1.06–7.81)**	**0.03035**
Cough and Sore throat	**2.24** (**1.26–3.97)**	**0.005051**
HCQ prophylaxis	0.77 (0.44–1.30)	0.3285

Next, we wanted to evaluate the IgG prevalence among symptomatic and asymptomatic HCWs. Around 38% of the studied population developed at least one of the COVID-19 symptoms in the recent past. We identified 24% of the asymptomatic HCWs developed IgG, whereas 52% of the symptomatic individuals were found to be seropositive for SARS-CoV-2 antibody (OR = 3.49, 95% CI = 2.14–5.70, *P*-value = 3.5 × 10^−7^) (Fig. 1D). We further evaluated the association of seropositivity among different COVID-19 suggestive symptoms, and found a significant association with all symptoms, among which fever and headache showed higher significance compared to other symptoms (OR = 3.397, 95% CI = 2.05–5.62, *P*-value = 1.179 × 10^−6^) (Table 2).

### Neutralising antibody sandwich ELISA

**Fig. 2.**
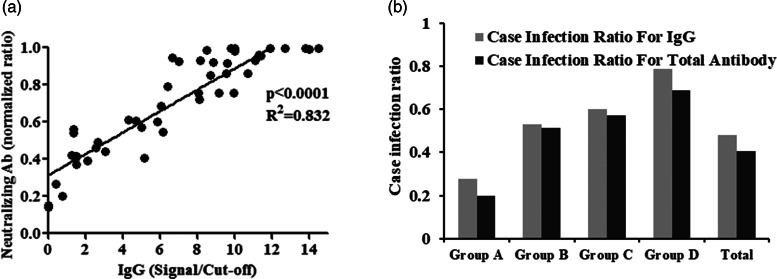
(A) Correlation between IgG and neutralising antibody titre (S/Co ratio). The line graph shows a significant positive correlation between IgG and neutralising antibody titre. (B) Case infection ratio among four occupational risk groups and all (total) samples.

To assess the extent of neutralising activity of the seropositive HCWs, we randomly selected 46 individuals from the IgG sero-reactive group and determined the level of neutralising antibody. The level of neutralising antibody was found to be positively correlated with the IgG S/Co ratio (*R*^2^ = 0.8363, *P*-value < 0.001) (Fig. 2A) suggesting that the IgG seropositive HCWs developed the protective antibody against SARS-CoV-2.

### Case infection ratio among HCWs

We further analysed the case detection using RT-PCR among the four groups of seropositive HCWs. Around 84% of RT-PCR positive HCWs showed detectable IgG, whereas 92% of the HCWs showed detectable level of total antibody. We determined the case-infection ratio for each group, where case refers to the RT-PCR tested COVID-19 positive cases and infection refers to the sero-reactive individuals in each group. Among the total samples, we found a case-infection ratio of 0.48 for IgG, suggesting that around half of the HCWs did not opt for the RT-PCR test (Fig. 2B). In group-wise analysis, a gradual increase of case-infection ratio from Group A to Group D was observed for both IgG and total antibody (Gr A = 0.27, B = 0.53, C = 0.60 and D = 0.78).

### Dynamics of anti-SARS-CoV-2 IgG antibody over time

**Fig. 3.**
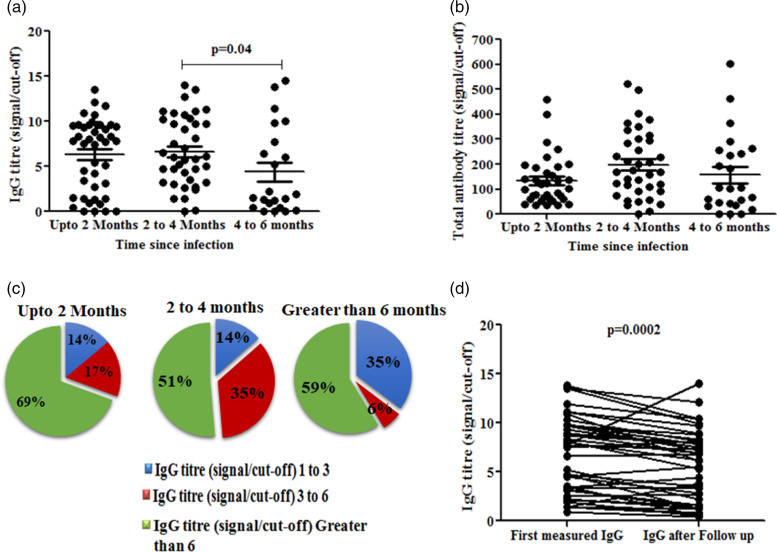
IgG dynamics among seropositive HCWs with time (A) IgG titre measured at 0–2 months, 2–4 months and 4–6 months post infection among RT-PCR confirmed COVID-19 cases. (B) Total antibody titre measured at 0–2 months, 2–4 months and 4–6 months post infection among RT-PCR confirmed COVID-19 cases. (C) Pie chart representing proportion of HCWs with IgG S/Co ratio at 0–2 months, 2–4 months and 4–6 months post infection among RT-PCR confirmed COVID-19 cases. (D) IgG titre (S/Co ratio) and the same after following up at 2 months interval.

To evaluate the antibody titre over time, we classified the RT-PCR confirmed COVID-19 HCWs with respect to time duration of COVID-19 RT-PCR testing to the first IgG measurement. The IgG and total antibody titres were found to be decreasing with times. The median values of IgG S/Co ratios were found to be 7.75 (MAD = 2.77), 5.97 (MAD = 3.21) and 1.70 (MAD = 1.685) at 0–2 months, 2–4 months and 4–6 months, respectively. Both IgG and total antibody titres were found to be significantly decreasing at 4–6 months post-infection (*P* = 0.04) (Fig. 3A, B). To get more insight about this trend, we classified HCWs into three groups having IgG titres of 1–3, 3–6 and >6 S/Co ratio and estimated the proportion of individuals within each group. Around 69% of HCWs showed IgG >6 S/Co ratio between 0–2 months and started decreasing at 2–4 months followed by a significant drop at 4–6 months post-infection (Fig. 3C). To substantiate this observation, we performed a follow up study with randomly selected 42 IgG seropositive individuals at 2 months interval. The IgG titre at 0–2 months showed a significant decrease at 2–4 months (*P* = 0.002) indicating the significant decreasing trend of IgG over time (Fig. 3D).

### Sero-reactivity after vaccination

**Fig. 4.**
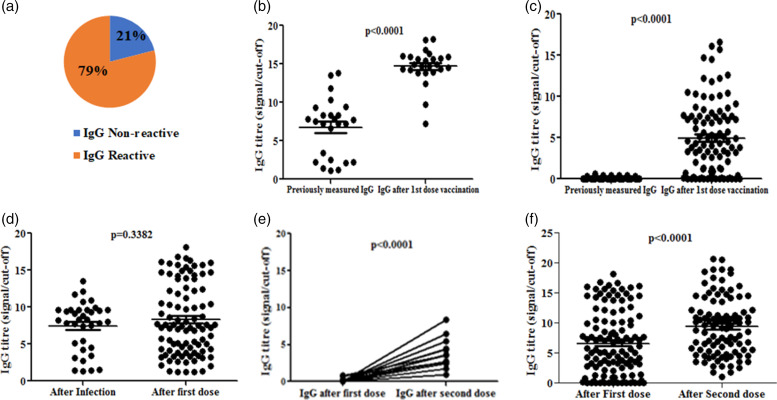
(A) Pie chart representing proportion of IgG reactive and non-reactive HCWs after first dose of vaccination. (B-C) IgG titre (S/Co ratio) among previously IgG (B) seropositive HCWs after first dose of vaccination and (C) seronegative HCWs after first dose of vaccination. (D) IgG titre (S/Co ratio) among naturally infected IgG seropositive HCWs and seronegative HCWs after first dose of vaccination. (E-F) IgG titre at 21 days' post first and second dose of vaccines among (E) seronegative and (F) seropositive HCWs after first dose vaccination.

Next, we evaluated the effect of vaccination on the development of anti-SARS-CoV-2 IgG. IgG titres of 119 HCWs after first dose and 99 HCWs after second dose of ChAdOx1 nCoV-19 vaccine were determined. Around 79% of the HCWs, who has taken first dose have developed detectable IgG, but surprisingly remaining 19% of them did not develop detectable amount of antibody against SARS-CoV-2 after 21 days from the first dose of vaccine (Fig. 4A). To better understand the antibody response among these non-reactive individuals, we categorised the first dose post-vaccination HCWs according to previous IgG status. It was observed that the IgG titre was significantly enhanced to 14.9 S/Co ratio (MAD = 1.49) after first dose of vaccination compared to previously seropositive (7.54 S/Co ratio (MAD = 3.02)) HCWs (*P* < 0.0001) (Fig. 4B). In case of previous seronegative HCWs, the median IgG was found to be 4.10 S/Co ratio (MAD = 3.53) after first dose of vaccination (Fig. 4C). Notably, the RT-PCR confirmed COVID-19 HCWs showed a median IgG titre of 8.25 S/Co ratio (MAD = 2.66) within 1–2 months post infection, indicating comparable effect of the first dose of vaccination (S/Co ratio = 7.48, (MAD = 4.07)) with that of the natural infection by SARS-CoV-2 (Fig. 4D). This observation clearly indicated that the first dose of vaccine among previously seropositive individuals acted as booster to enhance the level of antibody.

**Fig. 5.**
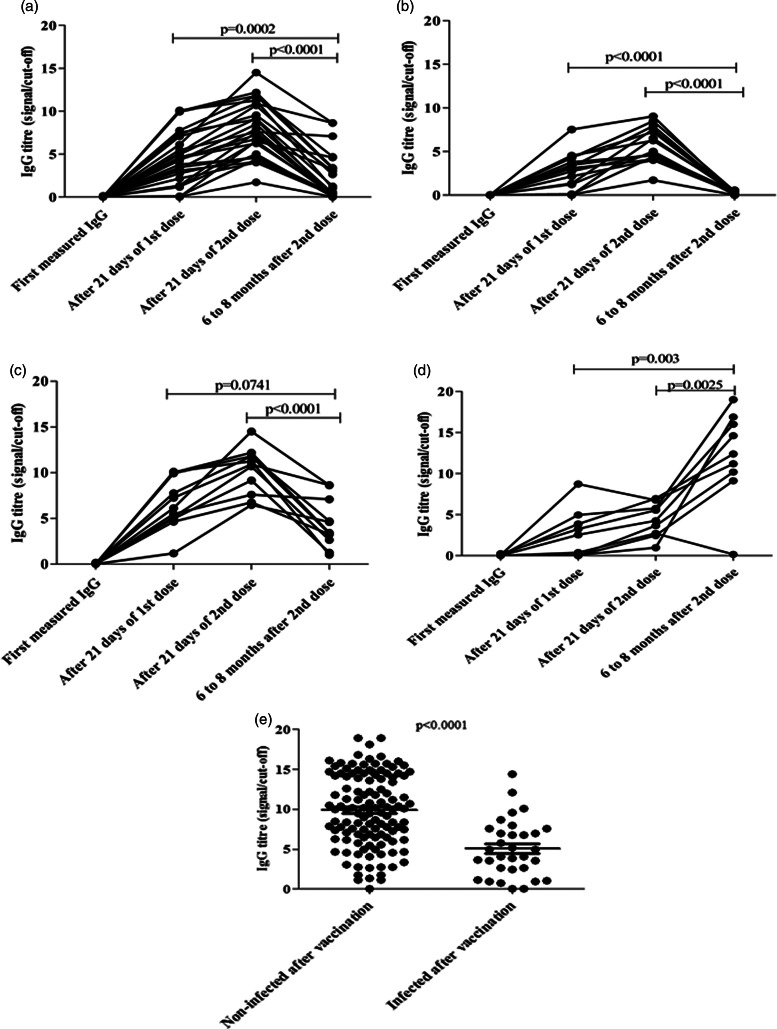
(A) The longitudinal data of IgG titres (S/Co ratio) of post vaccination non-infected HCWs from first dose to 6 to 8 months of second dose of vaccination. (B) The longitudinal data of IgG titres (S/Co ratio) of HCWs who encountered SARS-CoV-2 infection after any dose of vaccination from first dose to 6 to 8 months of second dose of vaccination. (C) The longitudinal data of IgG titres (S/Co ratio) of post vaccination non-infected seropositive HCWs from first measured to 6 to 8 months of second dose of vaccination. (D) IgG titre (S/Co ratio) among the vaccinated HCWs with or without SARS-CoV-2 infection within 6 to 8 months post vaccination. (E) IgG titre (S/Co ratio) among the breakthrough infected HCWs and uninfected HCWs.

Most of the vaccination strategy used a two-dose programme for the complete vaccination. To evaluate the antibody dynamics following two doses of vaccination, we determined the IgG of 119 HCWs after first dose and 99 HCWs after second dose of vaccine. Twenty-one days post first dose of vaccination, we found 19% HCWs remained seronegative while ~100% of them become seropositive after 21 days post second dose of vaccination, suggesting importance of second dose of vaccine (Fig. 4E). We further classified the HCWs as first dose non-reactive and reactive groups and determined antibody titre following 2 doses of vaccination. Interestingly, the non-reactive individuals after first dose of vaccination became seropositive to anti-SARS-CoV-2 IgG after the second dose (*P* = 0.0001) (Fig. 4E), whereas the IgG titre after second dose (S/Co ratio = 9.53, MAD = 4.03) significantly enhanced compared to first dose (S/Co ratio = 5.47, MAD = 4.57) (*P* < 0.0001) (Fig. 4F). Longitudinal data of anti-SARS-CoV-2 antibody replicated the general observation of significant enhancements after both first and second doses of ChAdOx1 nCoV-19 vaccine compared to the antibody titre before vaccination. Here we aimed to explore the status of anti-SARS-CoV-2 antibody titre after at least 6 months of second dose vaccination. We found that after 6 to 8 months of second dose vaccination the antibody titre significantly dropped where the HCWs didn't encounter any COVID infection after any dose of vaccination (S/Co ratio = 0.48, MAD = 0.45). After 6 to 8 months post vaccination and without any breakthrough infection during the time of observation, 56% of the HCWs didn't even have any detectable IgG (Fig. 5B) while the remaining also showed a significant drop in antibody titre compared to the second dose of vaccination (S/Co ratio = 3.43, MAD = 1.27) (Fig. 5C). On the other hand, we found HCWs who encountered breakthrough infection after any dose of vaccination still possessed a significant elevation of antibody titre after 6 to 8 months of second dose vaccination (Fig. 5D).

In our cohort of 313 HCWs, all of them received both doses of the ChAdOx1 nCoV-19 vaccine within December 2021. We measured the IgG titre following 21 days past first or second dose of vaccination for the 218 HCWs, and among them 153 HCWs were followed up for breakthrough infection until December 2021. We found that ~21% were infected with SARS-CoV-2 after any of the dose of post vaccination. Interestingly, ~89% of the infected HCWs showed either mild or no symptoms and recovered in home isolation without requirement of any O_2_ support. Only 11% HCW needed hospitalisation with moderate to severe symptoms and O_2_ requirement. To evaluate whether the level of antibody could serve as a possible indicator of post vaccination infection, we compared the median antibody titre among the post-vaccination uninfected and infected HCWs. The median antibody S/Co ratio for the infected HCWs (S/Co ratio = 4.51, MAD = 2.45) was significantly less compared to the uninfected HCWs (S/Co ratio = 10, MAD = 3.78) (*P* < 0.001) (Fig. 5E), suggesting a possibility of SARS-CoV-2 infection among the low antibody titre seropositive individuals. Note that other factors such as degree of precautionary measures, occupational risk factors among these groups might also be a perilous factor for such infection.

## Discussion

To understand the transmission dynamics of SARS-CoV-2 virus, seroprevalence is important to guide interventions for control of COVID-19 pandemic. In a tertiary care hospital set up, seroprevalence guides the COVID-19 infection dynamics among the different working groups [23]. As HCWs are the most exposed to SARS-CoV-2 infection, determination of seroprevalence among different working groups is an effective indicator to monitor and control the spread of SARS-CoV-2 infection. Seroprevalence rate was found to be related with various factors like selection bias of study participants, study period, awareness and effective implementation of infection control practices, use of PPE, maintenance of hand hygiene and physical distance, identification, isolation and quarantine as well as seroprevalence rate in the community [24–28].

In this study, the seropositivity among HCWs in a tertiary care hospital was 34% for IgG during November 2020 to January 2021, which was similar to a study conducted at a tertiary hospital in New York City, USA (36%) [29]. The seroprevalence among HCWs in our study was little higher to the reported seroprevalence of 26% among the HCWs in Kolkata during September 2020 [28]. The third pan India serosurvey, conducted during December 2020 – January 2021 also reported 25.6% seroprevalence among the HCWs in India [30]. In contrary to our observation, the pan India serosurvey did not identify any difference in seroprevalence between different HCW categories. This difference might be due to inadequate stratification of risk groups among the HCWs. However, variable prevalence among HCWs across different parts of the country might also impact the results of their study.

The overall seroprevalence data showed no significant association with age or sex, as also was observed in second and third pan India serosurvey conducted in August and December 2020 [30, 31]. Seropositivity of highly exposed two groups (Group A & B) was similar, whereas Group C and Group D showed significantly low seropositivity compared to these groups. The decreasing trend of seroprevalence ranging from 54% to 23% from high risk to low-risk group for both IgG and total antibody vividly portrayed the association of seroprevalence with SARS-CoV-2 infection risk. The total antibody titre (S/Co ratio) was significantly higher than IgG because it includes IgA, IgM along with IgG. Among the IgG non-reactive individuals, 10% showed the presence of detectable amount of total anti-SARS-CoV-2 antibody, suggesting the presence of only IgA and IgM. The pan India serosurvey also reported variable seroprevalence in urban and rural areas with higher prevalence in urban slum areas than rural areas [31].

Working in COVID-19 or non-COVID-19 unit was not associated with increased antibody positivity in HCWs. The seroprevalence study from a Spanish hospital also found no association of COVID-19 with working in COVID unit suggesting that awareness and strict adherence to infection control protocols were sufficient to prevent transmission to HCWs [25].

This study also evaluated the effect of HCQ as prophylaxis for COVID-19 in terms of seroprevalence among the HCQ consumers and non-consumers. In a hospital-based seroprevalence study in Kolkata, there was a significant association of sero-reactivity with adequate HCQ consumption as only 1.3% of HCQ consumers became reactive [28]. But in this study, we did not find any effect of HCQ prophylaxis on seroprevalence of COVID-19.

We know that the cellular immune response plays an important role to clear the virus from host cell and humoral immune response is responsible for preventing future infection. Although the plaque reduction neutralisation test (PRNT) is the gold standard for neutralisation assays, it is cumbersome, time consuming and require Biosafety Level 3 facilities. Therefore, we performed ELISA based neutralising assay to substantiate the preventive immunity of anti-spike IgG and found 100% of seropositive individuals developed detectable neutralising antibody. The signal cut-off values of detected IgG were directly proportional to the level of neutralising antibody.

We found a positive correlation of both IgG and total antibody reactivity with COVID-19 suggestive symptoms developed throughout the course of the disease as 52% of all symptomatic individuals were sero-reactive. So, early identification of suggestive symptoms acts as a predictive indication of SARS-CoV-2 infection and based on that self-isolation can be recommended to prevent the further spread. Seroprevalence study at Sweden showed that almost all COVID-19 symptoms were highly associated the sero-reactivity [26]. The first seroprevalence study at Belgium also found a significant association of COVID-19 symptoms with seroprevalence [23].

We have determined the RT-PCR positive cases among IgG positive HCWs as case infection ratio for each group in our cohort. The gradual increasing trend of case infection ratio from group A to group D decisively showed that most of the infections were undetected in HCWs from high-risk groups whereas the low-risk group HCWs were aware of their infection and did RT-PCR test in time.

The durability of the antibody titre depends on its initial titre and the time of measurement following infection or vaccination. We found that IgG titre was significantly reduced after 4 to 6 months of infection and the proportion of individuals in each IgG titre level decreased over time. A brief follow-up after 2 months, with randomly selected 42 seropositive individuals also substantiated the decay kinetics of IgG. This data was concomitant with a previous study, where it was shown that the titre could last up to 5 months [32]. A CDC report on November 2020 showed similar observations where 94% of 156 seropositive HCWs experienced a rapid decline in antibody titre after 60 days of initial observation [33].

The remarkable volume of knowledge accumulated from the scientific quest during this pandemic helped in yielding actionable insights which lead to develop vaccines and therapeutic strategies against COVID-19. As of now, 137 vaccine candidates are at various stages of clinical trials, and 194 vaccines are in preclinical development spanning diverse vaccination platforms [14]. Administration of adenovirus-based vaccines COVISHEILD was initiated in India for HCWs in early January, 2021 [15]. We systematically analysed the development of anti-spike glycoprotein IgG antibody after first and second dose of COVISHIELD vaccination. Startlingly, 19% of them did not develop detectable anti-spike IgG after 21 days of the first vaccination. We also found that, for previously seropositive HCWs, the first dose acted as a booster dose and the detectable IgG titre was significantly elevated than previous antibody level. In contrary, seronegative individuals also developed detectable IgG after the first dose. Although, there was a reduction in IgG titre in pre-vaccinated follow up measurements, it showed a significant increase after first dose of vaccination. A comparable median IgG (S/Co ratio = 8.25, MAD = 2.66) were observed among the RT-PCR tested positive and the previously sero-negative first dose vaccinated HCWs (S/Co ratio = 7.48, MAD = 4.07) suggested the effectiveness of first dose vaccination for antibody production. After administration of the second dose, ~100% of first dose seronegative individuals developed detectable IgG and the antibody titre was also significantly elevated. For the first dose seropositive individuals the IgG titre after the second dose was also significantly elevated. To check the resilience of the vaccine-induced antibody, we performed a strategic follow up after 6 to 8 months of second dose of vaccination. HCWs, who didn't face any breakthrough infection after second dose of vaccination, were significantly losing the vaccine-induced IgG titre and 56% of them didn't have detectable IgG. The IgG titre for the rest of the IgG reactive 44% individuals was also significantly lower than second dose of vaccination titre. This observation cumulatively suggests the need of booster dose for the HCWs in terms of vaccine-induced antibody production. Exposure to the SARS-CoV-2 among the HCWs is generally higher compared to other individuals due to their involvement in patient care, especially in a COVID-19 tertiary care centre. SARS-CoV-2 infections among the vaccinated HCWs were found to be ~21%, while the disease severity among them was very low compared to the unvaccinated HCWs. Although, in our study we did not investigate the protection of antibody after vaccination on newly emerged SARS-CoV-2 variants, the recent report suggests that the vaccination with ChAdOx1 nCoV-19 is as effective against the B.1.1.7 variant of SARS-CoV-2 as other lineages and results in reduction in viral load as well as duration of virus shedding, which obviously decrease the transmission of disease [34]. ChAdOx-1 nCov-19 vaccine showed 9-fold lower *in vitro* neutralisation activity against B.1.1.7 (double mutant strain) *vs*. a canonical non-B.1.1.7 strain [34]. This observation along with the enhanced antibody generation supports the immense potential of ChAdOx-1 nCov-19 vaccine. On the other hand, in a very recent report UK Health Security Agency (UKHSA) stated that a third booster of COVISHIELD vaccine provides 70% to 75% protection against symptomatic infection from B.1.1.529 variant (Omicron) [35]. To conclude, our study, which dealt with the anti-SARS-CoV-2 antibody dynamics starting from prevalence through follow-up up to the 6 to 8 months of second dose of vaccination and its association on several significant factors, may help to build better preventive strategies in future. Similar comprehensive study over the general population following vaccination will be necessary to monitor the trend and optimal resource utilisation for better management of the ongoing pandemic in a large country like India.

## Data Availability

Most of the data used here are presented in the manuscript. Other data supporting the results reported in this study will be available from the first author, Dr Soma Sarkar (drdssarkar@gmail.com) upon request after taking into consideration of ethical issues.
